# Spatial interplay of lymphocytes and fibroblasts in estrogen receptor-positive HER2-negative breast cancer

**DOI:** 10.1038/s41523-022-00416-y

**Published:** 2022-04-28

**Authors:** I. Nederlof, S. Hajizadeh, F. Sobhani, S. E. A. Raza, K. AbdulJabbar, R. Harkes, M. J. van de Vijver, R. Salgado, C. Desmedt, M. Kok, Y. Yuan, H. M. Horlings

**Affiliations:** 1 Division of Tumor Biology and Immunology, The Netherlands Cancer Institute, Amsterdam, The Netherlands; 2 Division of Molecular Pathology, The Netherlands Cancer Institute, Amsterdam, The Netherlands; 3 Centre for Evolution and Cancer, The Institute of Cancer Research, London, UK; 4 Division of Molecular Pathology, The Institute of Cancer Research, London, UK; 5 Department of Computer Science, University of Warwick, Coventry, UK; 6 Bioimaging Facility, The Netherlands Cancer Institute, Amsterdam, The Netherlands; 7 Department of Pathology, Amsterdam University Medical Centre, Amsterdam, The Netherlands; 8 Department of Pathology, GZA-ZNA Hospitals, Antwerp, Belgium; 9 Division of Clinical Medicine and Research, Peter MacCallum Cancer Centre, Melbourne, Australia; 10 Laboratory for Translational Breast Cancer Research, Department of Oncology, KU Leuven, Leuven, Belgium; 11 Division of Medical Oncology, The Netherlands Cancer Institute, Amsterdam, The Netherlands

**Keywords:** Breast cancer, Cancer microenvironment, Prognostic markers

## Abstract

In estrogen-receptor-positive, HER2-negative (ER^+^HER2^−^) breast cancer, higher levels of tumor infiltrating lymphocytes (TILs) are often associated with a poor prognosis and this phenomenon is still poorly understood. Fibroblasts represent one of the most frequent cells in breast cancer and harbor immunomodulatory capabilities. Here, we evaluate the molecular and clinical impact of the spatial patterns of TILs and fibroblast in ER^+^HER2^−^ breast cancer. We used a deep neural network to locate and identify tumor, TILs, and fibroblasts on hematoxylin and eosin-stained slides from 179 ER^+^HER2^−^ breast tumors (ICGC cohort) together with a new density estimation analysis to measure the spatial patterns. We clustered tumors based on their spatial patterns and gene set enrichment analysis was performed to study their molecular characteristics. We independently assessed the spatial patterns in a second cohort of ER^+^HER2^−^ breast cancer (*N* = 630, METABRIC) and studied their prognostic value. The spatial integration of fibroblasts, TILs, and tumor cells leads to a new reproducible spatial classification of ER^+^HER2^−^ breast cancer and is linked to inflammation, fibroblast meddling, or immunosuppression. ER^+^HER2^−^ patients with high TIL did not have a significant improved overall survival (HR = 0.76, *P* = 0.212), except when they had received chemotherapy (HR = 0.447). A poorer survival was observed for patients with high fibroblasts that did not show a high level of TILs (HR = 1.661, *P* = 0.0303). Especially spatial mixing of fibroblasts and TILs was associated with a good prognosis (HR = 0.464, *P* = 0.013). Our findings demonstrate a reproducible pipeline for the spatial profiling of TILs and fibroblasts in ER^+^HER2^−^ breast cancer and suggest that this spatial interplay holds a decisive role in their cancer-immune interactions.

## Introduction

The endogenous anti-cancer immune response is often expressed as the percentage of tumor infiltrating lymphocytes (TILs) and is tightly associated with a good prognosis in triple negative breast cancer (TNBC) patients^1–3^. In stark contrast with TNBC, high levels of TILs in estrogen receptor-positive, HER2 receptor-negative (ER^+^HER2^−^) breast cancer were associated with recurrence of disease^4^, clinico-pathological features of dismal outcome^5^, and were an adverse prognostic factor in several large clinical cohorts^1,6^. Intriguingly, TIL prognostic value seems different in ER^+^ patients treated with or without chemotherapy^5^ and there are also studies showing that a higher level of CD8 T cells is associated with a better outcome^7^. Automated analysis also revealed that increased spatial clustering of immune and cancer cells correlated with poor prognosis in ER^+^ breast cancer^8^, again in contrast with TNBC^9^. A clear explanation for this potentially opposite or diverse effect of TILs in ER^+^HER2^−^ breast cancer is still lacking.

Most studies on the spatial organization of breast cancer^8–12^ have focused only on TILs in the context of the tumor cells, often forgoing the role and interactions of other cells in the tumor that can alter the cancer-immune interactions. Accumulating pre-clinical and clinical evidence show that fibroblasts are key mediators in tumor structure and immunomodulation^13,14^. Fibroblast presence in breast tumors was linked to prognosis already two decades ago^15–17^, and recent breast cancer studies uncovered several subtypes of fibroblasts^13,14,18^, differently enriched in ER^+^HER2^−^ and TNBC tumors^13,19,20^. Therefore, fibroblasts potentially hold a decisive role in the potentially contrasting breast cancer-immune response in ER^+^HER2^−^ breast tumors. We hypothesized that if the presence and spatial distribution of fibroblasts in a tumor has an impact on TILs or vice versa, then these features can be used to define distinct spatial patterns which can potentially provide new insight into the cancer-immune response. Here we evaluate the patterns and clinical impact of the proximity between tumor, TILs, and fibroblast in ER^+^HER2^−^ breast cancer.

## Results

### Cell detection and classification using deep learning in breast cancer FFPE H&E slides

**Fig. 1:**
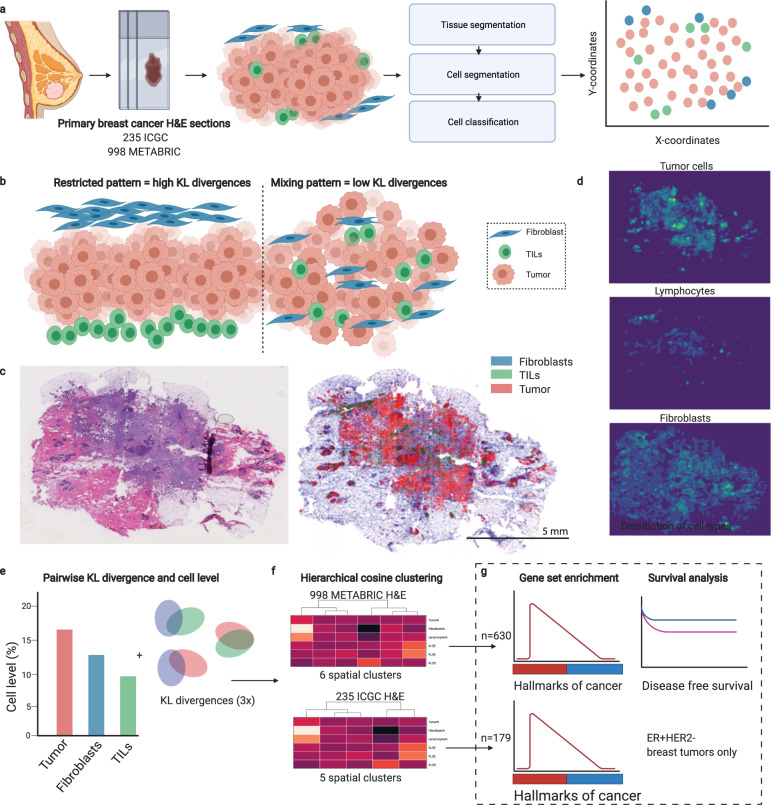
The analysis pipeline for automated image analysis and subsequent clustering of H&E slides. **a** Overview of the cohorts used and schematic illustration of the deep neural network-based pipeline for the processing of FFPE H&E slides. **b** Schematic examples of tumors with high KL-divergences (restricted patterns) and low KL-divergences (mixed patterns). **c** Example of FFPE H&E image and output of cell detection and classification. Scalebar indicates 5 mm. **d** Cell density distribution plots of H&E image under **c**. **e** Explanatory illustration of the three fractional levels of cell types, namely the cell type percentage of TILs, fibroblasts and tumor cells, and three measures to describe the mixing or restriction of the three different cell type distributions. The resulting six variables were used to cluster patients based on their H&E slides. **f** Hierarchical clustering of the patients in the ICGC cohort and METABRIC cohort separately. **g** Downstream analyses of molecular characteristics and survival of ER^+^HER2^−^ patients only.

**Fig. 2:**
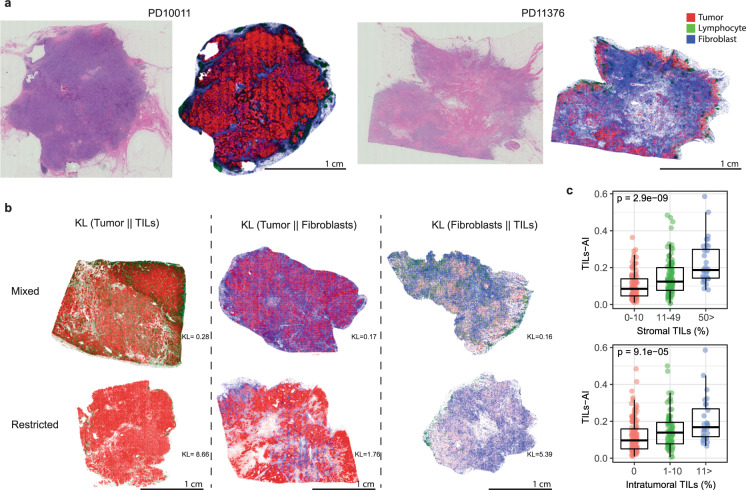
Cell detection and classification results of ICGC cohort. **a** Examples of the cell detection and classification results of 2 H&E images. **b** Examples images of H&E images with mixing or restriction of cell types. **c** The TILs-AI in ICGC breast tumors (*N* = 235) grouped by increasing levels of intra-tumoral or stromal TIL fraction (0–1) scored by pathologists (Kruskal–Wallis test). Scalebar for each image is 1 cm.

A deep neural network-based pipeline^21^ was used for detecting and classifying tumor cells, TILs, and fibroblasts from H&E slides from the ICGC breast cancer cohort^22^, which included 235 patients with an evaluable H&E slide from (T1–T3) primary breast cancer (Fig. 1a–d). For training of the cell classification model, in total 30,544 cells were annotated in 12 different FFPE H&E slides: 5553 TILs, 16,080 tumor cells, 4778 fibroblasts (including myofibroblasts) and 4133 other cells. The model reached an accuracy on average of 91.9% for the classification of cells (accuracy per cell type; Table S1). Cells detected with the deep neural network are hereafter referred to as *TILs-AI*, *tumor-AI*, and *fibro-AI*. As expected, in tumors with high stromal TILs or high intratumoral TILs percentages as assessed by pathologists, a higher TILs-AI level was detected (Fig. 2c, ICGC cohort, Fig. S1d R-sq = 0.176). In line with various immune cell estimates (TIL, cytotoxic T cells, B cells, regulatory T cells, fibroblasts; Fig. S1a, c) identified by standard pathology IHC^23^ and a RNA-based estimation on the ICGC cohort^24^ (Fig. S1a, c), the deep-learning model detected significantly lower TIL-AI levels in ER^+^ tumors versus ER^-^ tumors (10.5% vs. 17.9% resp., *P* = 0.019, Fig. S1b) and an overall higher fibro-AI levels in ER^+^ tumors compared to ER^−^ tumors (28.4% vs. 22.4% resp., *P* = 0.016 by Wilcoxon-rank test, Fig. S1b). As expected, tumor-AI levels were equal between ER^+^ and ER^−^ tumors (58.3% vs. 60.0%, *P* = 0.99 by Wilcoxon-rank test, Fig. S1b). The occurrence of a fibrotic scar on the H&E slides did not interfere with the overall % of TILs-AI or Fibro-AI (Fig. S1d, e). In summary, we observe a high accuracy of the model and find cell levels similar to classic pathology and RNA-based estimations in ER^+^ and ER^−^ breast tumors.

### Clustering of patients based on spatial architecture of H&E slides

**Fig. 3:**
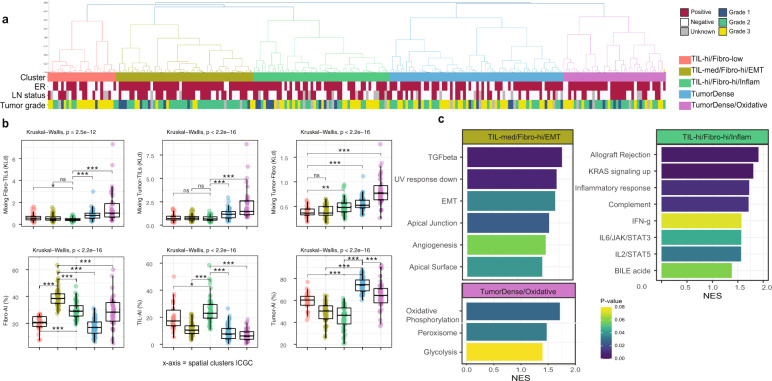
Hierarchical spatial clustering of ICGC breast tumors results in biological distinct groups. **a** Dendrogram of the hierarchical clustering of the ICGC cohort (*N* = 235) with annotated clinical parameters. **b** Boxplots of the six spatial H&E measurements that were used for the clustering. Box plots show median, lower, and upper hinges correspond to the 25th and 75th percentile. *Y*-axis shows KLd (three upper graphs) and percentages (three lower graphs). Kruskal–Wallis test and Wilcoxon-rank test for comparing the highest infiltrated or most mixed cluster to the rest. *P < 0.05; **P < 0.01; ***P < 0.001; ****P < 0.0001; NS not significant by Wilcoxon-rank test. **c** The normalized enrichment score (NES) for the hallmarks of cancer gene sets for each of the five spatial clusters (ER^+^HER2^−^ samples only, *N* = 179).

We expected that if the fibro-AI spatial distribution in a tumor has an impact on TILs-AI (or vice versa), then these features can be used to cluster breast tumors according to common spatial patterns of these cells. Example images from the ICGC cohort with the detection of cell types and corresponding Kullback–Leibler (KL) divergences are portrayed in Fig. 2a, b. The KL divergences and level of cell types were used to define tumor spatial clusters (Table 1 and Fig. 3a).

**Table 1:** Summary Table of Spatial Clusters.

Spatial cluster	Tumor	Fibro	TILs	Tumor-TILs	Fibro-TILS	Tumor-Fibro	GSEA
TIL-hi/Fibro-low	Moderate	Low	High	Mixing	Mixing	Mixing	None significant
Fibro-hi/EMT	Low	Highest	Moderate	+/−	Mixing	Mixing	EMT/TGF-β/Angiogenesis
TIL-hi/Fibro-hi/Inflamm	Lowest	High	Highest	Mixing	Mixing++	+/−	Inflammation/IFN-γ/Allograft/IL2-STAT5/IL6-JAK-STAT3
TumorDense	High	Lowest	Low	Restricted	Restricted	Restricted	None significant
TumorDense/Oxidative	Highest	Moderate	Lowest	Restricted	Restricted	Restricted	Oxidative phosphorylation, Peroxisome

GSEA analysis was performed with hallmarks of cancer gene sets (*N* = 50) for ER^+^HER2^−^ tumors only (*N* = 173).

*ER* estrogen receptor, *IFN-γ* interferon gamma, *TGF-β* tumor growth factor beta, *IL6* interleukin 6, *EMT* epithelial to mesenchymal transition, +/− not specifically mixed or restricted.

We identified three clusters with relatively high infiltration of TILs and/or fibroblasts and two clusters that were mainly packed with tumor cells. ER negative, basal-like, grade 3 tumors were commonly characterized by the TIL-high/Fibro-low cluster (Table S2). The majority of the tumors in the Fibro-hi/EMT cluster were Luminal A breast cancers, whilst the TumorDense and TumorDense/Oxidative clusters consisted mainly of luminal B breast cancer (Table S2). The Tables S2 and S3 suggest that some clinical characteristics are in fact unevenly distributed over the spatial clusters, but when we study the cell levels and spatial measures in subgroup analysis (Fig. S2), we observe that each subgroup (e.g., ER^+^ and ER^−^ subgroups) within the same spatial cluster follow the same patterns. This indicates that e.g., an ER^+^ and ER^−^ tumor in the same spatial cluster indeed shows similar cell infiltration, but the cluster itself may be enriched for ER^+^ breast tumors.

### Gene set enrichment of spatial clusters for ER^+^HER2^-^ patients

To understand if TILs in ER^+^HER2^−^ breast tumors were spatially linked to fibroblasts, we analyzed the spatial clusters for enrichment of the 50 hallmarks of cancer gene sets (Table 1 and Fig. 1g). We identified two TIL-high clusters, that differ greatly in fibroblast infiltration and spatial overlap. Patients with the highest TILs-AI levels were in the TIL-hi/Fibro-hi/Inflam cluster (median 23% vs 10.5% all ER^+^ samples) and significant enrichment of gene signatures like inflammatory response and allograft rejection were characteristic for these tumors (Fig. 3c). In contrast, the TIL-hi/Fibro-low cluster did not show a significant enrichment for inflammatory signatures compared to the rest. This suggests that the combination of fibro-AI and TILs-AI may lead to a synergistic inflammatory environment in ER^+^HER2^−^ breast cancer. The mean tumor cell content in the ICGC cohort was 58.9%, whilst in the TIL-hi/Fibro-hi/Inflam cluster this was 44.8% and, in the TIL-hi/Fibro-low cluster 59.3%, potentially leading to a relative dilution of inflammatory gene expression by immune cells in the latter. The Fibro-hi/EMT cluster was characterized by high Fibro-AI levels, with an upregulation of gene signatures like EMT, hypoxia, and TGF-ß signaling suggestive of fibroblast meddling (Fig. 3c). The two last clusters showed a relatively high tumor-AI percentage (Fig. 3b), of which one cluster (TumorDense/Oxidative) was enriched for oxidative phosphorylation and peroxisome gene sets, suggesting an altered oxidation in these tumors (Fig. 3c).

### Independent assessment of the spatial clusters in METABRIC

**Fig. 4:**
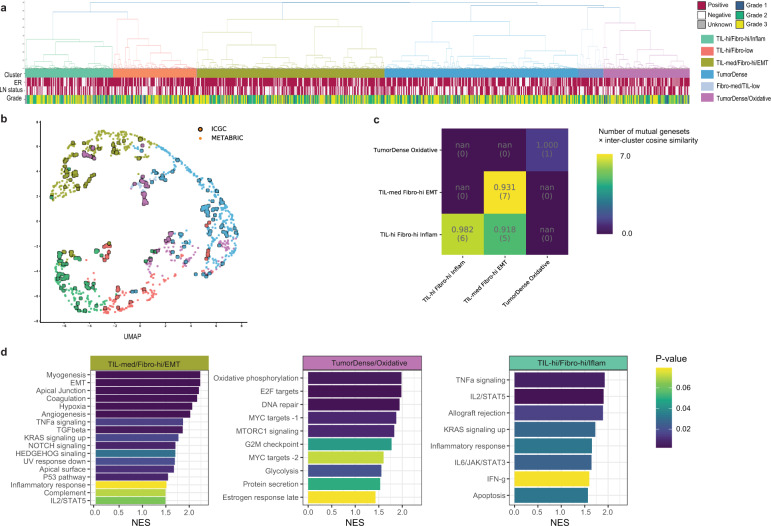
Spatial clusters in METABRIC cohort similar to ICGC cohort. **a** Dendrogram of the hierarchical clustering of the METABRIC cohort (*N* = 998) with annotated clinical parameters. **b** Two-dimensional representation of the six measurements that is acquired by applying a UMAP transformation for the ICGC and METABRIC clusters combined. **c** Heatmap of the cosine similarity of the mutual gene sets between clusters of the ICGC and METABRIC cohort, FDR *q*-value < 0.25. **d** The normalized enrichment score (NES) for the hallmarks of cancer gene sets for each of the six spatial clusters in METABRIC (ER^+^HER2^−^ samples only, *N* = 630).

If the spatial clustering of ER^+^HER2^−^ breast cancer leads to biologically distinct clusters, then these spatial clusters should be reproducible in another independent cohort. For this purpose, we studied the METABRIC cohort, where 997 H&E slides of primary breast cancer patients are available combined with overall survival data to study prognosis. The majority of samples were from ER^+^HER2^−^ patients (*N* = 630, mean age 61.4 years) of whom most received hormonal therapy (*N* = 528) and a smaller subgroup chemotherapy (*N* = 108). We used the cell type classification of samples in the METABRIC cohort (*N* = 997)^25^ and independently clustered them using the same analysis pipeline (Fig. 1). This resulted naturally in six clusters (Fig. 4a), one (small) extra cluster compared to ICGC. We observe the following similarities between the spatial clusters of ICGC and METABRIC; 1) similar cell distributions are observed per cluster (Fig. S2) 2) clusters localize together in the UMAP-based dimensionality reduction to the spatial measurements (Fig. 4b) and 3) the spatial clusters correlate for similar significantly enriched gene sets (Fig. 4c). We conclude that the analysis pipeline and clustering process (Fig. 1) is consistent and coherent when independently repeated over an independent cohort.

When performing GSEA for all ER^+^HER2^−^ (*n* = 630) samples based on the six spatial clusters of METABRIC, we found similar gene sets upregulated in specific clusters as in the ICGC cohort (Fig. 4d). The Fibro-hi/EMT cluster is again significantly enriched for EMT, hypoxia, and TGF-β. Interestingly, cluster TIL-hi/Fibro-lo and TIL-hi/Fibro-hi/Inflam both showed abundant TILs-AI infiltration (Fig. S2), however in line with the ICGC cohort only the cluster TIL-hi/Fibro-hi/Inflam showed significant enrichment for inflammatory gene sets (Fig. 4d). Last, we again find a TumorDense/Oxidative cluster, with significant enrichment for oxidative phosphorylation, and in addition estrogen and MYC targets, DNA repair and glycolysis.

### Prognostic value of the spatial clusters for ER^+^HER2^−^ patients

**Fig. 5:**
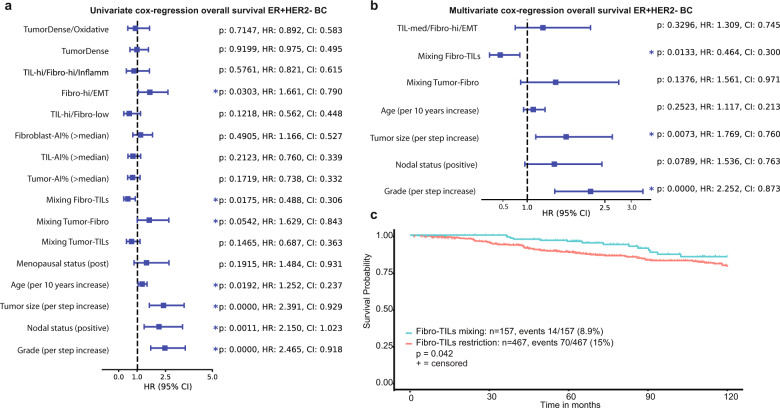
Spatial characteristics of ER^+^HER2^−^ breast cancer show prognostic value. **a** Univariate cox regression. Hazard ratios (HR) with 95% confidence intervals (CI) for the spatial measurements and clinical parameters for ER^+^HER2^+^ samples only (*N* = 603). *P*-values non-adjusted for multiple testing. **b** The multivariate cox regression for the significant univariate variables under **a** Hazard ratios (HR) with 95% confidence intervals (CI) on *x*-axis. **c** The overall survival probability of ER^+^HER2^−^ tumors with a low ${{{{D}}}}_{{{{\mathrm{KL}}}}}({{{{P}}}}_{{{{\mathrm{Fibroblast}}}}}||{{{{P}}}}_{{{{\mathrm{TILs}}}}})$ (=mixed) and high ${{{{D}}}}_{{{{\mathrm{KL}}}}}({{{{P}}}}_{{{{\mathrm{Fibroblast}}}}}||{{{{P}}}}_{{{{\mathrm{TILs}}}}})$ (=restricted). *P* values under **a** and **b** are the result of the log likelihood ratio test.

Last, we hypothesized that if combining the spatial measures and the cell ratios lead to reproducible spatial clusters, these will be associated with outcome. Only the METABRIC cohort includes clinical outcome, and therefore we first performed the univariate analyses of the spatial and clinical characteristics for ER^+^HER2^−^ tumors (*N* = 603, Fig. 5a). In Fig. S5, we show how the prognostic value of the TILs-AI changes with each 10th percentile step increase for ER^+^ and ER^+^HER2^−^ samples. In short, a TILs-AI cut-off value of p40–p60 shows a significant link to prognosis for ER^+^ patients, but not ER^+^HER2^−^ patients (Fig. S5). ER^+^HER2^−^ patients with a TILs-AI > p10 have a significantly lower HR than the rest (<p10) (HR = 0.443, *P* = 0.0323, log likelihood ratio (LLR) test). For all subsequent survival analysis we chose the cut-off value p50 to dichotomize the entire group equally into TIL-low or TIL-high.

We observe that a high TIL (>p50) is not significantly correlated with a better prognosis (HR = 0.760, *P* = 0.212, LLR test) for patients with ER^+^HER2^−^ breast cancer, also not when independently assessed in Luminal A (*N* = 273) and Luminal B (*N* = 193) breast cancer patients (Fig. S3). However, when we studied the prognostic role of the spatial analysis for the ER^+^HER2^−^ patients that received any form of chemotherapy, high TILs-AI level (>median) was linked to a significant better outcome (HR = 0.447, Fig. S4). In the univariate cox regression, Luminal B patients in the TIL-hi/Fibro-low clusters showed a significantly lower hazard ratio (0.226, *P* = 0.0405, LLR test). H&E image measurements that were correlated to a different overall survival for ER^+^HER2^−^ patients (*N* = 603) were the spatial mixing of fibro-AI and TILs-AI distributions ($D_{{\mathrm{KL}}}(P_{{\mathrm{Fibroblast}}}||P_{{\mathrm{TILs}}})$) (HR = 0.488, *P* = 0.0175, LLR test), spatial mixing of Tumor-AI and Fibro-AI ($D_{{\mathrm{KL}}}(P_{{\mathrm{Tumor}}}||P_{{\mathrm{Fibro}}})$ (HR = 1.629, *P* = 0.0542, LLR test) and the Fibro-hi/EMT spatial cluster (HR = 1.61, *P* = 0.0303, LLR test). Finally, we combined all univariate significant variables into a multivariate model (Fig. 5b). In the multivariate model only tumor grade, tumor size and mixing of fibro-AI and TILs-AI ($D_{{\mathrm{KL}}}\left( {P_{{\mathrm{Fibroblast}}}||P_{{\mathrm{TILs}}}} \right)$) remained significant contributors, indicating that the spatial interplay between fibroblasts and TILs may be important for clinical outcome in ER^+^HER2^−^ breast cancer patients (Fig. 5c).

## Discussion

Both TILs and fibroblasts are critical cells in shaping the tumor micro-environment and the clinical course of breast cancer patients^1,2,13,14,16–18^. Our understanding of how fibroblasts and TILs spatially form patterns that could alter the cancer immune-response is still lacking. Here, we use an innovative analysis to study the spatial patterns of fibroblasts, TILs, and tumor cells on H&E slides from primary breast tumors. Integrating cell abundance and patterns leads to a new classification of ER^+^HER2^−^ breast cancer that is associated with distinct molecular characteristics and our results indicate that the spatial organization of TILs and fibroblasts is consistent between independent cohorts and reproducible in both FFPE and FF samples.

In this study, ER^+^HER2^−^ breast cancer patients with high TIL did not show an improved or decreased survival (HR = 0.76, *P* = 0.21), in contrast with the negative effect on clinical outcome observed in other studies^1,6^. In line with a recent clinical study^5^, we showed that a higher level of TILs-AI was linked to a lower hazard ratio for ER^+^HER2^−^ patients that received chemotherapy. Intriguingly, we detected a cluster of ER^+^HER2^−^ tumors with high TILs-AI and fibro-AI levels with good spatial mixing, and inflammatory molecular characteristics, contradicting the previously observed negative reciprocity and immune exclusion of fibroblast and TILs^13^. However, patients with high TIL, high fibroblasts and high inflammatory gene signatures did not have an improved overall survival (HR = 0.821, *P* = 0.576, LLR test). This was unexpected, as tumors with inflammatory gene signaling are often considered immunologically active^26^, potentially leading to a better clinical outcome. It is plausible that the immunoregulatory activity of fibroblasts^14,27^ inhibit T-cell activation in these patients and counter-balance the inflammatory potential.

ER^+^HER2^−^ breast cancer patients in the cluster Fibro-hi/EMT showed a significantly poorer survival (HR = 1.661, *P* = 0.0303, LLR test). The enrichment of TGF-β signaling in this cluster hints potentially towards an immunosuppression strategy^27^, although from this current study we cannot draw the conclusion which cell type is responsible for the TGF-β secretion. However, in the multivariate analysis, only the mixing of TILs and Fibroblasts remained a significant contributor. This indicates that the spatial clusters identify molecular distinct subgroups with specific gene signaling, but prognostic value was mainly attributed to the spatial interplay of TILs and fibroblasts.

Whilst we have demonstrated that the spatial interplay of fibroblasts and TILs can be used to define molecular distinct subgroups in ER^+^HER2^−^ breast cancer, we do recognize limitations in our study.

First of all, working with H&E slides offers the advantage of easy accessibility and uniformity against the disadvantage of the difficulty of phenotyping subtypes of cells. TILs can easily be detected based on morphology, however, which type of TILs are present cannot currently be deduced from the H&E slide. This is even more evident for fibroblasts, which can take many sizes and shapes, leaving the possibility that we systematically miss a certain subtype of fibroblasts. The variable interplay between TILs and fibroblasts in the spatial clusters may originate from the specific phenotypes of TILs and fibroblasts in these tumors^14,18,28,29^. Future studies that combine (multiplexed) immunohistochemistry with matched H&E deep learning could offer new insights, which subtle cell subtypes could be recognized from the H&E slides and which cell types are systematically biased.

When introducing a new method to study the immune environment, it is vital to place this into context of already available methodologies. Computational classification and analysis of the H&E slides has the advantage of speed and scalability. However, it is important to acknowledge that currently we cannot assume our computational TILs score is the same as the stromal sTIL score from pathologists. Comparison of the automated TILs scores with a pathologist’s sTILs score^2^ in the ICGC showed a weak correlation overall, in line with previous studies that compared the computational TILs to pathology TILs^8^. This is unsurprising when one considers that the automated scores include regions of the tumor that are excluded on pathological evaluation, and thus the information distilled from these methods potentially provides different biological information. On top of the fact that computational TILs may give different biological information, there are currently different ways on how to use the TILs scores as they have been categorized in various manners for clinical evaluation, either dichotomized^30^ or e.g., in three subgroups^1^. Our current simple dichotomization of the samples in low versus high can potentially be too simplistic.

Last, the prognostic value of the spatial measurements has been tested on one retrospective cohort that was not primarily focused on the analysis of the H&E images; therefore, these results should be considered as hypothesis generating and need to be validated in independent clinical cohorts.

In conclusion, our findings demonstrate a reproducible pipeline for the spatial profiling of breast tumors from H&E slides, and showed that the spatial interplay of fibroblasts and TILs potentially hold a decisive role in the ER^+^HER2^−^ breast cancer-immune response. Future research should clarify if this can truly aid in patient stratification or treatment optimization.

## Methods

### Patient cohorts and tissue sections

Two hundred and fifty-eight Hematoxyline and Eosine (H&E) slides containing breast cancer tissue of patients included in the ICGC cohort^22^ were subjected to fully automated image analysis for single-cell classification at a resolution of 20x magnification. Out of those, 235 H&E slides were included for downstream analysis after excluding images with low quality (e.g., large artifacts or no invasive component), of which 179 were of ER^+^HER2^−^ tumors^22^. Two pathologists scored the infiltrate on the ICGC H&E stained slides for stromal TILs and intratumoral TILs according to existing guidelines^2,23^. In addition, we used all 998 available Fresh Frozen (FF) H&E slides from the METABRIC cohort (*N* = 630 ER^+^HER2^−^ tumors) to study the spatial patterns^25,31^. HER2 status for METABRIC was previously scored via IHC for 442/998 samples. Samples with an IHC-HER2 score of 0 and 1 were categorized as HER2-negative. Samples with an IHC-HER2 score of 3 were categorized as HER2-positive. Samples with an IHC-HER2 score of 2 or unknown were categorized based on the amplification status of the HER2, samples with a gain were categorized as HER2-positive^25,31^). Of the 753 ER+ samples, 630 samples were HER2-negative and 123 samples HER2-positive (Table S6).

Lymphocyte infiltration in the METABRIC cohort was scored during central pathology review for a subset of the patients (*N* = 359 with H&E slide for digital analysis)^31^, not using the more recent guidelines for TILs^2^ and thus were categorized into “absent”, “mild”, and “severe” (included in Table S3). This study was approved by the local ethics committee at the Netherlands Cancer Institute (CFMPB54) and we complied with all relevant ethical regulations.

### The cell detection model and spatial scatter

We used a deep neural network-based cell detection and classification model pipeline consisting of three parts: (I) tissue segmentation, (II) cell segmentation and (III) cell classification. Training and testing of the model were performed over a 5-fold cross validation. The annotations were randomly divided into five equal groups. Samples used for training of the model are listed in Table S7. Class imbalance was taken into account while creating these five groups. For each cross-validation, four groups were chosen for training/validation and one group for testing. Out of the four groups of training/validation data, 20% of the annotations was randomly picked for validation and the remaining data was used to train the network.

(I) Tissue segmentation removes background reducing the amount of noise and artefacts in the data which in turn allows for a more computationally efficient cell segmentation and classification with increased accuracy^21^. Tissue segmentation was performed using a pre-trained Micro-Net-508 model which was trained earlier on randomly selected 100 lung slides from TCGA. The accuracy of tissue segmentation remained above 97% for all the manually annotated samples in ICGC H&E slides and therefore we did not retrain the model.
(II) Secondly, the cell segmentation model was trained using the Micro-Net-252 algorithm^32^ on a combined cohort of ConSep^33^, CPM-15, CPM-17 and Kumar data set^34^ to segment the cells in the tissue regions. The network visualizes the image at multiple resolutions, captures context information by connecting intermediate deep layers and adds bypass connections to max-pooling to maintain weak features. This led to a robust segmentation of tissue regions and cells in the presence of noise.
(III) Lastly, a cell classification framework utilizes a neighboring ensemble predictor classifier to classify each cell [SCCNN-TMI2016]^35^. This predictor utilizes Inception-V3 network instead of SCCNN network to classify cells in neighboring locations to the detected center (centroid) of the segmented cell. In our implementation, the ensemble classifier required votes from Inception-V3 classification of nine different neighborhood locations near to the center of the cell compared to five votes in ensemble predictor implementation of SCCNN. Through experimentation, the patch size was optimized to 51 × 51 for classification instead of 27 × 27 as originally proposed. A dedicated breast pathologist (H.M.H) annotated the slides for tumor cells, fibroblasts, lymphocytes and other cells i.e., nerve cells, endothelial cells, red blood cells, and fat cells.

To analyze the spatial distribution of the different cell subtypes, pixel coordinates were taken for each of the three main types of cells: tumor cells, lymphocytes, and fibroblasts, and were used to fit a 2-dimensional non-parametric Kernel Density Estimator (KDE) with a Gaussian kernel (Fig. 1c, d). The size of the kernel bandwidth parameters was determined based on our assumption that the effective range of a lymphocyte cell within the tumor microenvironment is approximately 50 microns (up to five cell layers away). Over each slide, three KDEs are therefore fitted to the positions of the detected three cell type distributions. The fitted density distributions are then evaluated on a grid of points that cover the entire tumor tissue space while leaving the rest out, to make the measurement insensitive to the size of the tumor tissue and non-tumor tissue. Figure 1 shows an example of the detected cell positions (Fig. 1c) as well as the measured densities of each cell type using the fitted density estimators (Fig. 1d).

To quantify the proximity of the pairs of cell type distributions, we measure the Kullback–Leibler divergence^36^ (Eq. 1) between the 3 pairs of cell type distributions. The lower the Kullback–Leibler divergence is, the more similar the distribution of that specific cell type is to the distribution of the target cell type (Fig. 2b).

1 $$D_{{\mathrm{KL}}}\left( {P_{\mathrm{X}}||Q_{\mathrm{X}}} \right) = {\int}_{ - \infty }^\infty {dX{\mathrm{p}}(X){\mathrm{Log}}\left( {\frac{{P(X)}}{{Q(X)}}} \right)}$$

In Eq. 1*X* represents the 2-dimensional space of the positions of the cells that are acquired using the cell detection model. We calculate $D_{{\mathrm{KL}}}(P_{{\mathrm{Tumor}}}||P_{{\mathrm{TILs}}})$, $D_{{\mathrm{KL}}}(P_{{\mathrm{Tumor}}}||P_{{\mathrm{Fibroblast}}})$, and $D_{{\mathrm{KL}}}(P_{{\mathrm{Fibroblast}}}||P_{{\mathrm{TILs}}})$ using the per type cell density estimators.

To study relative spatial mixing or restriction of two cell types, e.g., TILs-AI and tumor-AI, we classified patients <1st quartile of $D_{{\mathrm{KL}}}(P_{{\mathrm{Tumor}}}||P_{{\mathrm{TILs}}})$, as mixing and patients >1st quartile of $D_{{\mathrm{KL}}}(P_{{\mathrm{Tumor}}}||P_{{\mathrm{TILs}}})$, as spatially restricted.

It is worth mentioning that the asymmetricity of the Kullback–Leibler divergence, e.g., $D_{{\mathrm{KL}}}(A||B)$ is not equal to $D_{{\mathrm{KL}}}\left( {B||A} \right)$, is a desirable behavior. The intention is to quantify similarities of the distribution of one cell type, for instance, lymphocytes relative to another target cell type such as tumor cells. Therefore, how the lymphocytes are distributed outside the tumor area is not meant to be affecting this measure. The lower the Kullback–Leibler divergence is, the more similar the distribution of that specific cell type is to the distribution of the target cell type (Fig. 2b).

### Clustering of spatial measures

In total, we have six measurements per slide; the ratios of tumor cells, lymphocytes and fibroblasts within the tumor area and the proximity measures of the following pairs of cell types $D_{{\mathrm{KL}}}(P_{{\mathrm{Tumor}}}||P_{{\mathrm{TILs}}})$, $D_{{\mathrm{KL}}}(P_{{\mathrm{Tumor}}}||P_{{\mathrm{Fibroblast}}})$, and $D_{{\mathrm{KL}}}(P_{{\mathrm{Fibroblast}}}||P_{{\mathrm{TILs}}})$. To create the clustering, we start by creating the linkage matrix between all 235 samples in the ICGC breast cancer cohort^22^. Cosine distance^37,38^ is used as the measure of distance. The reason for this choice is to emphasize the relative size of the six measurements rather than their absolute values. This is especially preferable because the units are different among KL measures and the frequency ratios, and therefore the Euclidean-like distances could introduce measurement interpretation error. Once the linkage matrix is calculated, hierarchical clustering was used to group the samples together. We tested different linkage rules and decided to choose the complete linkage-clusters that binds sub-clusters with their furthest samples being compared, to acquire a more evenly distributed set of clusters and observing no single nodes. Using the resulting dendrogram (Fig. 3a), we picked the threshold that splits samples into five clusters for the ICGC cohort. By applying the same set of steps to H&E slides from our validation cohort METABRIC, the dendrogram there naturally splits into six clusters (Fig. 4a). To relate the clusters between the two cohorts, we calculated the basic statistics of the six measurements of each of the clusters across the two cohorts (Fig. S2).

### Transcriptomic data

RNA sequencing data was available for 184 out of 235 patients from the ICGC breast cancer cohort^22,39^ for whom H&E slides were available and 998 patients from the METABRIC cohort^31^. For the ICGC cohort, the published transcriptome was used and transformed to transcripts per million (tpm) and log2 transformed. We used the molecular subtypes^40^ as previously published^39^. To infer immune cell populations from transcriptomic data, MCP-counter^24^ was used. For gene set enrichment analysis between two categorical groups (e.g., cluster [X] versus all other clusters), the GSEA software version 4.1.0 of the Broad Institute with the hallmarks of cancer (*N* = 50) from MsigDB^41^ were used. Detailed gene set information is available on the Broad Institute at [http://www.gsea-msigdb.org/gsea/msigdb/genesets.jsp?collection=H]. For all gene set analysis, we used the ER^+^HER2^−^ samples only. For METABRIC, Human_Illumina_HumanWG_6_v3_MSigDB.v7.1.chip was used. We set the gene set size filters (min = 15, max = 500) and used 1000 permutations per comparison. The statistical output tables for the ICGC and METABRIC GSEA are available in Tables S4 and S5. The normalized enrichment scores (NES) and FDR *q*-values from the GSEA were subsequently used for visualizations in Figs. 3 and 4.

### Statistics and survival analysis

Statistical analysis was performed in R version 4.0.2 and Python V3. Correlation was carried out with the Spearman nonparametric rank correlation test. Nonparametric Wilcoxon–Mann–Whitney tests were applied for comparisons between two different groups. Kruskal–Wallis test by ranks was performed for comparisons of all 3+ groups. *P* values were considered significant if less than 0.05, and significance values were corrected for multiple testing by FDR for the gene set enrichment analysis. *χ*^2^ contingency test was used to test for imbalances in proportions of clinical parameters between clusters.

The endpoint of interest was overall survival in ER^+^HER2^−^ breast cancer patients. Overall survival was defined as the date of death from any cause. We used the Kaplan–Meier method to establish survival curves and the log-rank test to compare survival curves across subgroups. To test the association between the spatial variables, cluster results and prognosis in ER^+^HER2^−^ breast cancer, we used univariate ad multivariate Cox proportional hazards models. For the survival analysis, patients were split according to whether they were above or below the median of cellular levels. For the KL divergences, patients were split according to whether they were below the first quartile (mixed distribution) or above the first quartile (restricted distribution). For the spatial clusters, each cluster was compared against the rest, for example (cluster B) vs. (all clusters [A:E] – cluster B). Following the univariate analysis, we constructed a multivariate Cox proportional hazards model. The model variables are known clinical predictors, including lymph node metastasis (positive vs. negative), menopausal status (pre vs. post), age (increasing steps of 10 years), tumor size (one step increase of tumor size; e.g., T1:T2 or T2:T3), tumor grade (one step increase in grade; e.g., grade 1:grade 2 or grade 2:grade 3) and the significant spatial variables from the univariate analysis.

## Supplementary information

Supplementary figures and tables

Table S4

Table S5

## Data Availability

Access to data of the ICGC cohort^22^ was granted by the International Cancer Genome Consortium (ICGC) under DACO#6329. Clinical and transcriptomic data are controlled and available at the centralized METABRIC and ICGC repositories (https://docs.icgc.org/; https://ega-archive.org/dacs/EGAC00001000484).

## Code availability

Code used for the spatial analysis of H&E slides, clustering, gene set enrichment figures, UMAP plot and survival analysis are available from https://codeocean.com/capsule/0775202/tree (all code and data available there to instantly acquire results). The statistics in this study were computed using R (version 4.0.2) or Python 3. The *χ*^2^ contingency tests were calculated using Scipy.stats (https://www.scipy.org/). The cell detection and classification were implemented using TensorFlow (https://www.tensorflow.org/) in python. The kernel density estimations and the clustering analysis were done in Python with standard function from Scipy (https://www.scipy.org/) and Scikit-learn (https://scikit-learn.org/) packages. All plots were produced with R (https://ggplot2.tidyverse.org/) and Python (https://matplotlib.org/). All survival analysis were performed in Python V3 with the survival package (https://lifelines.readthedocs.io) with the CoxPHfitter function for the cox regression analysis. All software dependencies are available in the Comprehensive Repository R Archive Network (CRAN; https://cran.r-project.org/), Bioconductor (https://www.bioconductor.org/), or The Broad Institute GSEA (https://www.gsea-msigdb.org/gsea/index.jsp).

## References

[1] Denkert C (2018). Tumour-infiltrating lymphocytes and prognosis in different subtypes of breast cancer: a pooled analysis of 3771 patients treated with neoadjuvant therapy. Lancet Oncol..

[2] Salgado R (2015). The evaluation of tumor-infiltrating lymphocytes (TILs) in breast cancer: recommendations by an International TILs Working Group 2014. Ann. Oncol..

[3] Park, J. H. et al. Prognostic value of tumor-infiltrating lymphocytes in patients with early-stage triple-negative breast cancers (TNBC) who did not receive adjuvant chemotherapy. *Ann. Oncol*. **30**, 1941–1949 (2019). 10.1093/annonc/mdz395 31566659

[4] Sobral-Leite M (2019). Cancer-immune interactions in ER-positive breast cancers: PI3K pathway alterations and tumor-infiltrating lymphocytes. Breast Cancer Res..

[5] Criscitiello C (2020). Tumor-infiltrating lymphocytes (TILs) in ER+/HER2- breast cancer. Breast Cancer Res. Treat..

[6] Ali HR (2014). Association between CD8+ T-cell infiltration and breast cancer survival in 12,439 patients. Ann. Oncol..

[7] Krijgsman, D. et al. Quantitative whole slide assessment of tumor-infiltrating CD8-positive lymphocytes in ER-positive breast cancer in relation to clinical outcome. *IEEE J. Biomed. Heal. Inform.* **25**, 1–1 (2020). 10.1109/JBHI.2020.3003475 32750943

[8] Heindl A (2018). Relevance of spatial heterogeneity of immune infiltration for predicting risk of recurrence after endocrine therapy of ER+ breast cancer. J. Natl Cancer Inst..

[9] Nawaz S, Heindl A, Koelble K, Yuan Y (2015). Beyond immune density: critical role of spatial heterogeneity in estrogen receptor-negative breast cancer. Mod. Pathol..

[10] Saltz J, Gupta R, Hou L, Lazar AJ, Sharma A (2018). Spatial organization and molecular correlation of tumor-infiltrating lymphocytes using deep learning on pathology images. Cell Rep..

[11] Maley CC, Koelble K, Natrajan R, Aktipis A, Yuan Y (2015). An ecological measure of immune-cancer colocalization as a prognostic factor for breast cancer. Breast Cancer Res..

[12] Yuan, Y. Modelling the spatial heterogeneity and molecular correlates of lymphocytic infiltration in triple-negative breast cancer. *J. R. Soc. Interface* **12**, 20141153 (2015). 10.1098/rsif.2014.1153 PMC4305416 25505134

[13] Costa A (2018). Fibroblast heterogeneity and immunosuppressive environment in human breast cancer. Cancer Cell.

[14] Kieffer Y (2020). Single-cell analysis reveals fibroblast clusters linked to immunotherapy resistance in cancer. Cancer Discov..

[15] Orimo A (2005). Stromal fibroblasts present in invasive human breast carcinomas promote tumor growth and angiogenesis through elevated SDF-1/CXCL12 secretion. Cell.

[16] Chang, H. Y. et al. Gene expression signature of fibroblast serum response predicts human cancer progression: Similarities between tumors and wounds. *PLoS Biol*. **2**, e7 (2004). 10.1371/journal.pbio.0020007 PMC314300 14737219

[17] Chang HY (2005). Robustness, scalability, and integration of a wound-response gene expression signature in predicting breast cancer survival. Proc. Natl Acad. Sci. USA.

[18] Friedman G (2020). Cancer-associated fibroblast compositions change with breast cancer progression linking the ratio of S100A4+ and PDPN+ CAFs to clinical outcome. Nat. Cancer.

[19] Ali HR (2020). Imaging mass cytometry and multiplatform genomics define the phenogenomic landscape of breast cancer. Nat. Cancer.

[20] Jackson, H. W. et al. The single-cell pathology landscape of breast cancer. *Nature* **578**, 615–620 (2020). 10.1038/s41586-019-1876-x 31959985

[21] AbdulJabbar, K. et al. Geospatial immune variability illuminates differential evolution of lung adenocarcinoma. *Nat. Med*. **26**, 1–9 (2020). 10.1038/s41591-020-0900-x PMC7610840 32461698

[22] Nik-Zainal S (2016). Landscape of somatic mutations in 560 breast cancer whole-genome sequences. Nature.

[23] Nederlof I (2019). Comprehensive evaluation of methods to assess overall and cell-specific immune infiltrates in breast cancer. Breast Cancer Res..

[24] Becht E (2016). Estimating the population abundance of tissue-infiltrating immune and stromal cell populations using gene expression. Genome Biol..

[25] Yuan Y (2012). Quantitative image analysis of cellular heterogeneity in breast tumors complements genomic profiling. Sci. Transl. Med..

[26] Rooney MS, Shukla SA, Wu CJ, Getz G, Hacohen N (2015). Molecular and genetic properties of tumors associated with local immune cytolytic activity. Cell.

[27] Mariathasan S (2018). TGFβ attenuates tumour response to PD-L1 blockade by contributing to exclusion of T cells. Nature.

[28] Gruosso T (2019). Spatially distinct tumor immune microenvironments stratify triple-negative breast cancers. J. Clin. Invest..

[29] Egelston, C. A. et al. Resident memory CD8+ T cells within cancer islands mediate survival in breast cancer patients. *JCI Insight* **4**, 19 (2019). 10.1172/jci.insight.130000 PMC6795408 31465302

[30] Loi S (2019). Tumor-infiltrating lymphocytes and prognosis: a pooled individual patient analysis of early-stage triple-negative breast cancers. J. Clin. Oncol..

[31] Curtis C (2012). The genomic and transcriptomic architecture of 2000 breast tumours reveals novel subgroups. Nature.

[32] Raza SEA (2019). Micro-Net: a unified model for segmentation of various objects in microscopy images. Med. Image Anal..

[33] Graham, S. et al. Hover-Net: simultaneous segmentation and classification of nuclei in multi-tissue histology images. *Med. Image Anal*. **58**, 101563 (2019). 10.1016/j.media.2019.101563 31561183

[34] Kumar N (2017). A dataset and a technique for generalized nuclear segmentation for computational pathology. IEEE Trans. Med. Imaging.

[35] Sirinukunwattana K (2016). Locality sensitive deep learning for detection and classification of nuclei in routine colon cancer histology images. IEEE Trans. Med. Imaging.

[36] Kullback S, Leibler RA (1951). On information and sufficiency. Ann. Math. Stat..

[37] Singhal, A. Modern information retrieval: a brief overview. IEEE Data Eng. Bull. **24**, 35–43 (2001).

[38] Popat, S. K., Deshmukh, P. B. & Metre, V. A. Hierarchical document clustering based on cosine similarity measure. in *Proceedings - 1st International Conference on Intelligent Systems and Information Management, ICISIM 2017* 153–159 (Institute of Electrical and Electronics Engineers Inc., 2017).

[39] Smid, M. et al. Breast cancer genome and transcriptome integration implicates specific mutational signatures with immune cell infiltration. *Nat. Commun*. **7**, 1–9 (2016). 10.1038/ncomms12910 PMC5052682 27666519

[40] Perou CM (2000). Molecular portraits of human breast tumours. Nature.

[41] Subramanian A (2005). Gene set enrichment analysis: a knowledge-based approach for interpreting genome-wide expression profiles. Proc. Natl Acad. Sci. USA.

