# Supplementary material for: Spatial interplay of lymphocytes and fibroblasts in estrogen receptor-positive HER2-negative breast cancer
Source: NPJ Breast Cancer. 2022 Apr 28;8:56. doi: 10.1038/s41523-022-00416-y (PMC9051105; doi:10.1038/s41523-022-00416-y)
Supplement: Supplementary file 1 — Supplementary figures and tables [file 41523_2022_416_MOESM1_ESM.pdf]

Supplementary Figures and Tables

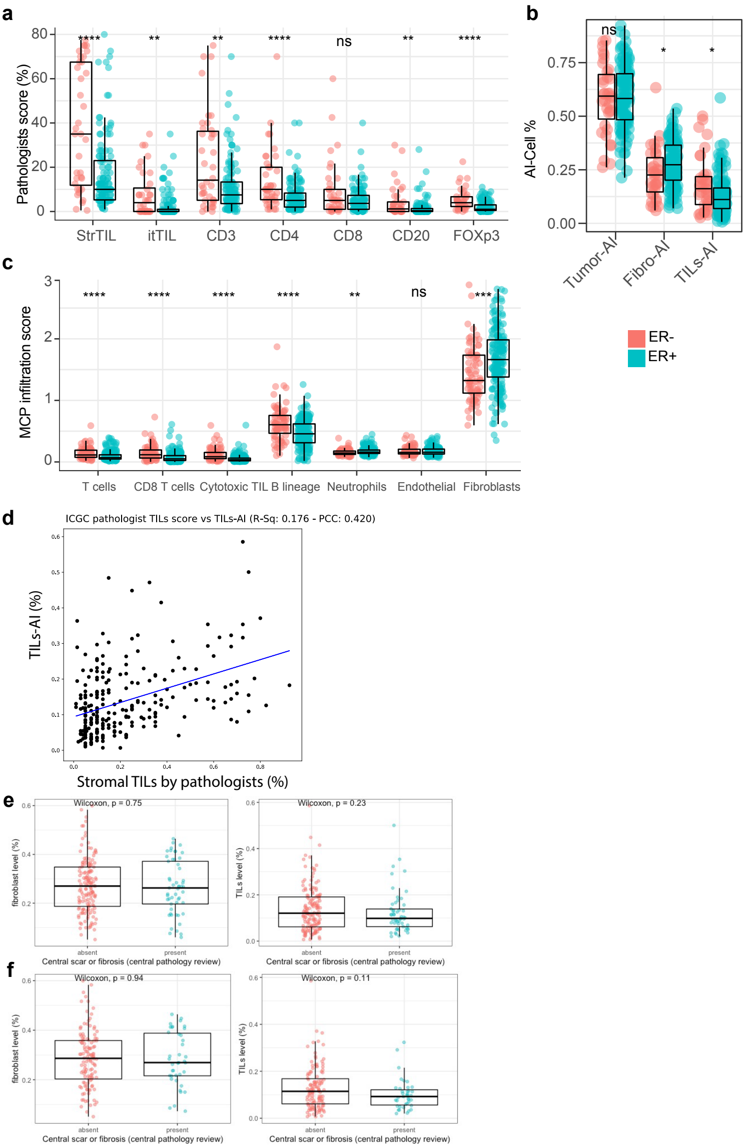
a
Pathologists score (%)
StrTIL
itTIL
CD3
CD4
CD8
CD20
FOXp3
****
**
**
****
ns
**
****
b
AI-Cell %
Tumor-AI
Fibro-AI
TILs-AI
ns
*
*
ER-
ER+
c
MCP infiltration score
T cells
CD8 T cells
Cytotoxic TIL
B lineage
Neutrophils
Endothelial
Fibroblasts
****
****
****
****
**
ns
***
d
ICGC pathologist TILs score vs TILs-AI (R-Sq: 0.176 - PCC: 0.420)
TILs-AI (%)
Stromal TILs by pathologists (%)
e
Wilcoxon, p = 0.75
fibroblast level (%)
absent
present
Central scar or fibrosis (central pathology review)
Wilcoxon, p = 0.23
TILs level (%)
absent
present
Central scar or fibrosis (central pathology review)
f
Wilcoxon, p = 0.94
fibroblast level (%)
absent
present
Central scar or fibrosis (central pathology review)
Wilcoxon, p = 0.11
TILs level (%)
absent
present
Central scar or fibrosis (central pathology review)

Supplementary Figure 1. Lymphocytes and fibroblasts in the tumor microenvironment of ER^+ and ER^- breast cancer (ICGC cohort).

(a) Lymphocyte levels in ER^+ (N=179) and ER^- (N =56) breast tumors scored by pathologists on H&E-stained slides or immunohistochemically stained. **(b)** Cell proportions as a result from the deep learning model for tumor-AI, fibro-AI and TILs-AI, separated by ER status. **(c)** Immune cells levels calculated by MCP-counter in ER+ and ER- breast tumors. **(d)** R-sq between TILs-AI and sTILs scored by pathologists. **(e)** TILs-AI and Fibro-AI levels (fraction 0-1) in all ICGC samples with and without a fibrotic scar as scored on central pathology review. **(f)** TILs-AI and Fibro-AI levels (fraction 0-1) in all ICGC ER^+ samples (n=179) with and without a fibrotic scar as scored on central pathology review. (*P < 0.05; **P < 0.01; *** P < 0.001; **** P < 0.0001; NS, not significant by Wilcoxon-rank test. All boxplots show median, lower and upper hinges correspond to the 25th and 75th percentile.

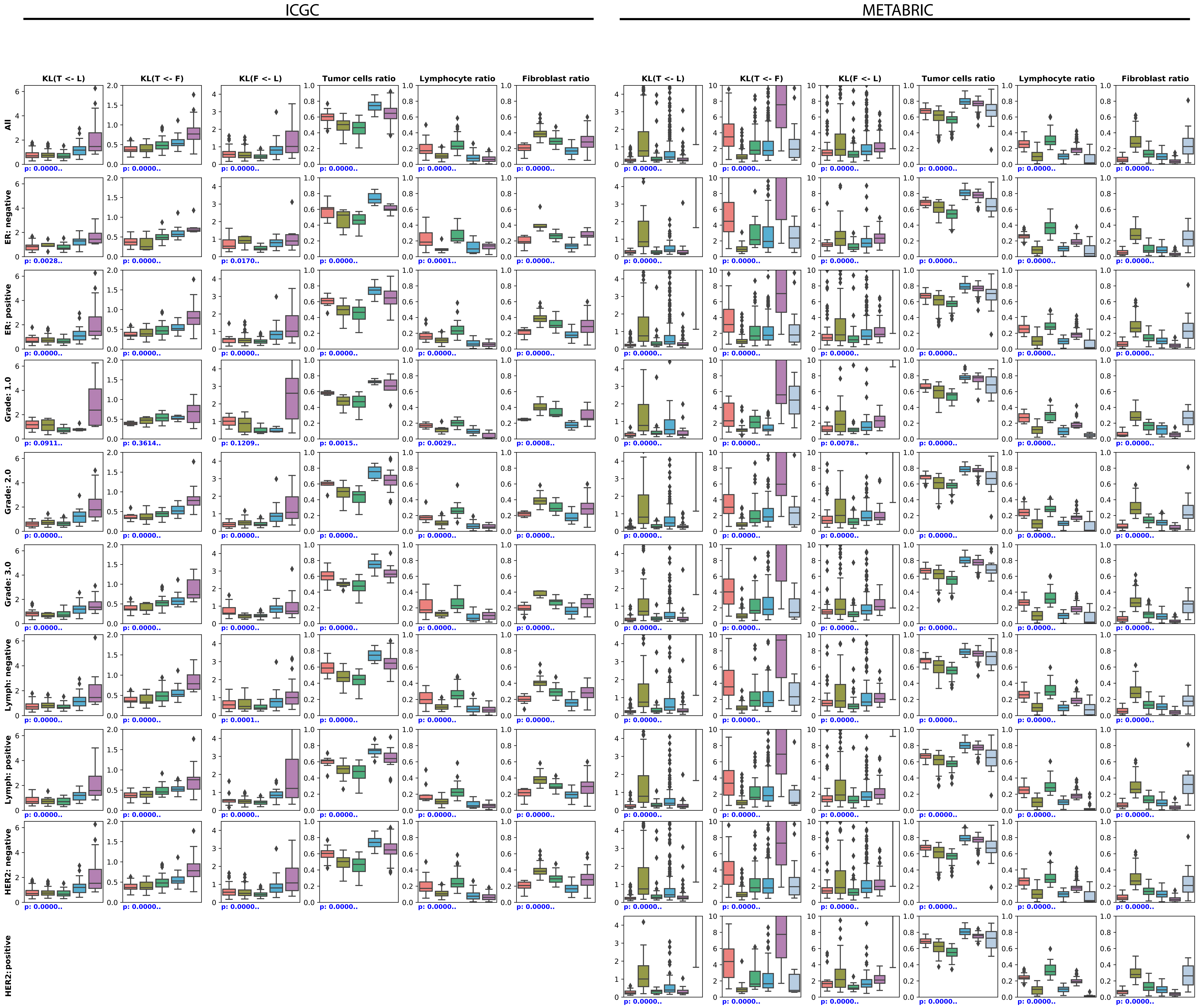

ICGC
METABRIC
KL(T <- L)
KL(T <- F)
KL(F <- L)
Tumor cells ratio
Lymphocyte ratio
Fibroblast ratio
All
ER: negative
ER: positive
Grade: 1.0
Grade: 2.0
Grade: 3.0
Lymph: negative
Lymph: positive
HER2: negative
HER2: positive

Supplementary Figure 2. The boxplots for ICGC (left) and METABRIC (right) for the 6 measures (horizontal) used in the model for all samples, and stratified according to clinical parameters (vertical), including ER status, HER2 status, lymph node status and grade. Boxplots show median, lower and upper hinges correspond to the 25th and 75th percentile. All P-values by Wilcoxon-rank test.

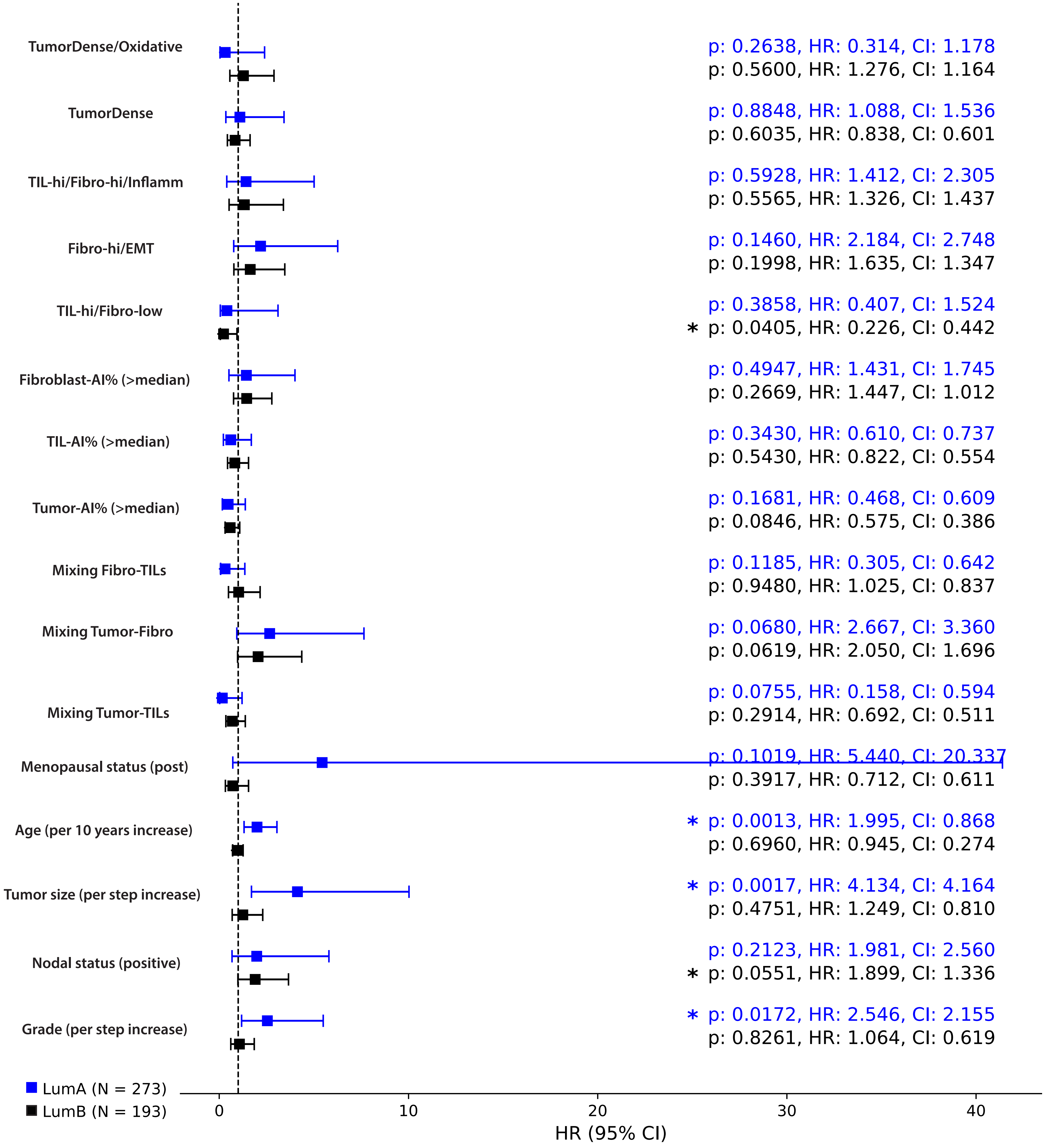
TumorDense/Oxidative
p: 0.2638, HR: 0.314, CI: 1.178
p: 0.5600, HR: 1.276, CI: 1.164
TumorDense
p: 0.8848, HR: 1.088, CI: 1.536
p: 0.6035, HR: 0.838, CI: 0.601
TIL-hi/Fibro-hi/Inflamm
p: 0.5928, HR: 1.412, CI: 2.305
p: 0.5565, HR: 1.326, CI: 1.437
Fibro-hi/EMT
p: 0.1460, HR: 2.184, CI: 2.748
p: 0.1998, HR: 1.635, CI: 1.347
TIL-hi/Fibro-low
p: 0.3858, HR: 0.407, CI: 1.524
* p: 0.0405, HR: 0.226, CI: 0.442
Fibroblast-AI% (>median)
p: 0.4947, HR: 1.431, CI: 1.745
p: 0.2669, HR: 1.447, CI: 1.012
TIL-AI% (>median)
p: 0.3430, HR: 0.610, CI: 0.737
p: 0.5430, HR: 0.822, CI: 0.554
Tumor-AI% (>median)
p: 0.1681, HR: 0.468, CI: 0.609
p: 0.0846, HR: 0.575, CI: 0.386
Mixing Fibro-TILs
p: 0.1185, HR: 0.305, CI: 0.642
p: 0.9480, HR: 1.025, CI: 0.837
Mixing Tumor-Fibro
p: 0.0680, HR: 2.667, CI: 3.360
p: 0.0619, HR: 2.050, CI: 1.696
Mixing Tumor-TILs
p: 0.0755, HR: 0.158, CI: 0.594
p: 0.2914, HR: 0.692, CI: 0.511
Menopausal status (post)
p: 0.1019, HR: 5.440, CI: 20.337
p: 0.3917, HR: 0.712, CI: 0.611
Age (per 10 years increase)
* p: 0.0013, HR: 1.995, CI: 0.868
p: 0.6960, HR: 0.945, CI: 0.274
Tumor size (per step increase)
* p: 0.0017, HR: 4.134, CI: 4.164
p: 0.4751, HR: 1.249, CI: 0.810
Nodal status (positive)
p: 0.2123, HR: 1.981, CI: 2.560
* p: 0.0551, HR: 1.899, CI: 1.336
Grade (per step increase)
* p: 0.0172, HR: 2.546, CI: 2.155
p: 0.8261, HR: 1.064, CI: 0.619
LumA (N = 273)
LumB (N = 193)
0
10
20
30
40
HR (95% CI)

Supplementary Figure 3. Single variate cox regression for LumA and LumB ER+HER2- patients in the METABRIC cohort.

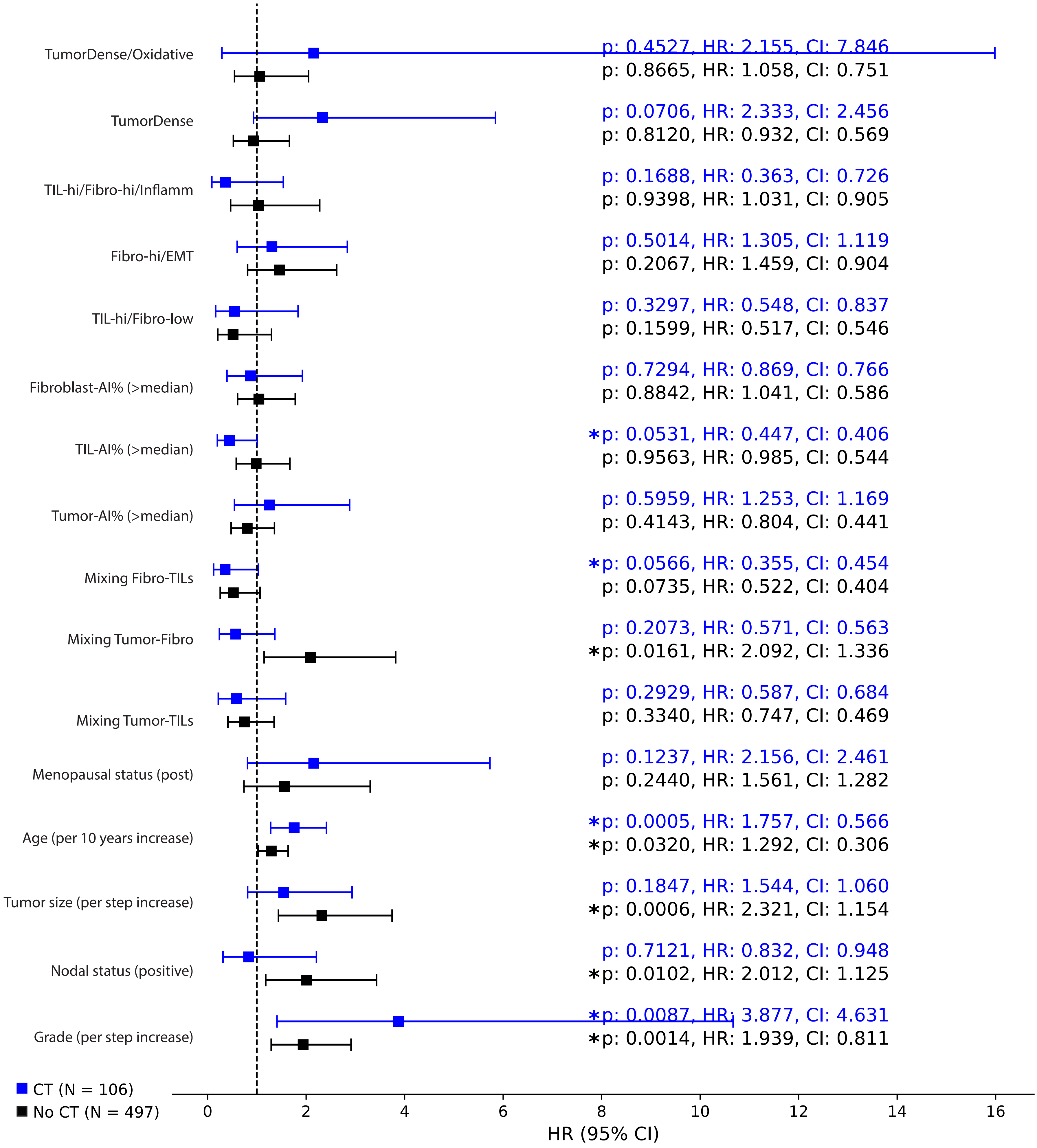

TumorDense/Oxidative
p: 0.4527, HR: 2.155, CI: 7.846
p: 0.8665, HR: 1.058, CI: 0.751
TumorDense
p: 0.0706, HR: 2.333, CI: 2.456
p: 0.8120, HR: 0.932, CI: 0.569
TIL-hi/Fibro-hi/Inflamm
p: 0.1688, HR: 0.363, CI: 0.726
p: 0.9398, HR: 1.031, CI: 0.905
Fibro-hi/EMT
p: 0.5014, HR: 1.305, CI: 1.119
p: 0.2067, HR: 1.459, CI: 0.904
TIL-hi/Fibro-low
p: 0.3297, HR: 0.548, CI: 0.837
p: 0.1599, HR: 0.517, CI: 0.546
Fibroblast-AI% (>median)
p: 0.7294, HR: 0.869, CI: 0.766
p: 0.8842, HR: 1.041, CI: 0.586
TIL-AI% (>median)
*p: 0.0531, HR: 0.447, CI: 0.406
p: 0.9563, HR: 0.985, CI: 0.544
Tumor-AI% (>median)
p: 0.5959, HR: 1.253, CI: 1.169
p: 0.4143, HR: 0.804, CI: 0.441
Mixing Fibro-TILs
*p: 0.0566, HR: 0.355, CI: 0.454
p: 0.0735, HR: 0.522, CI: 0.404
Mixing Tumor-Fibro
p: 0.2073, HR: 0.571, CI: 0.563
*p: 0.0161, HR: 2.092, CI: 1.336
Mixing Tumor-TILs
p: 0.2929, HR: 0.587, CI: 0.684
p: 0.3340, HR: 0.747, CI: 0.469
Menopausal status (post)
p: 0.1237, HR: 2.156, CI: 2.461
p: 0.2440, HR: 1.561, CI: 1.282
Age (per 10 years increase)
*p: 0.0005, HR: 1.757, CI: 0.566
*p: 0.0320, HR: 1.292, CI: 0.306
Tumor size (per step increase)
p: 0.1847, HR: 1.544, CI: 1.060
*p: 0.0006, HR: 2.321, CI: 1.154
Nodal status (positive)
p: 0.7121, HR: 0.832, CI: 0.948
*p: 0.0102, HR: 2.012, CI: 1.125
Grade (per step increase)
*p: 0.0087, HR: 3.877, CI: 4.631
*p: 0.0014, HR: 1.939, CI: 0.811
CT (N = 106)
No CT (N = 497)
0
2
4
6
8
10
12
14
16
HR (95% CI)

Supplementary Figure 4. Single variate cox regression for patients that received chemotherapy or no chemotherapy in the METABRIC cohort.

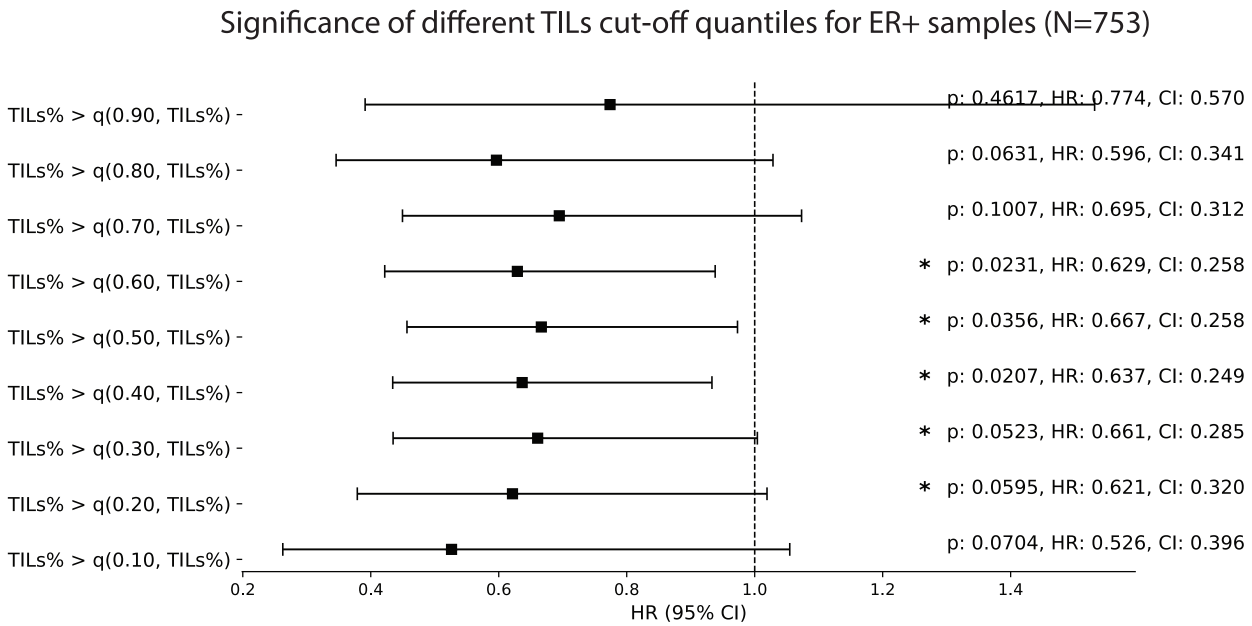
Significance of different TILs cut-off quantiles for ER+ samples (N=753)
TILs% > q(0.90, TILs%)
TILs% > q(0.80, TILs%)
TILs% > q(0.70, TILs%)
TILs% > q(0.60, TILs%)
TILs% > q(0.50, TILs%)
TILs% > q(0.40, TILs%)
TILs% > q(0.30, TILs%)
TILs% > q(0.20, TILs%)
TILs% > q(0.10, TILs%)
p: 0.4617, HR: 0.774, CI: 0.570
p: 0.0631, HR: 0.596, CI: 0.341
p: 0.1007, HR: 0.695, CI: 0.312
* p: 0.0231, HR: 0.629, CI: 0.258
* p: 0.0356, HR: 0.667, CI: 0.258
* p: 0.0207, HR: 0.637, CI: 0.249
* p: 0.0523, HR: 0.661, CI: 0.285
* p: 0.0595, HR: 0.621, CI: 0.320
p: 0.0704, HR: 0.526, CI: 0.396
0.2
0.4
0.6
0.8
1.0
1.2
1.4
HR (95% CI)

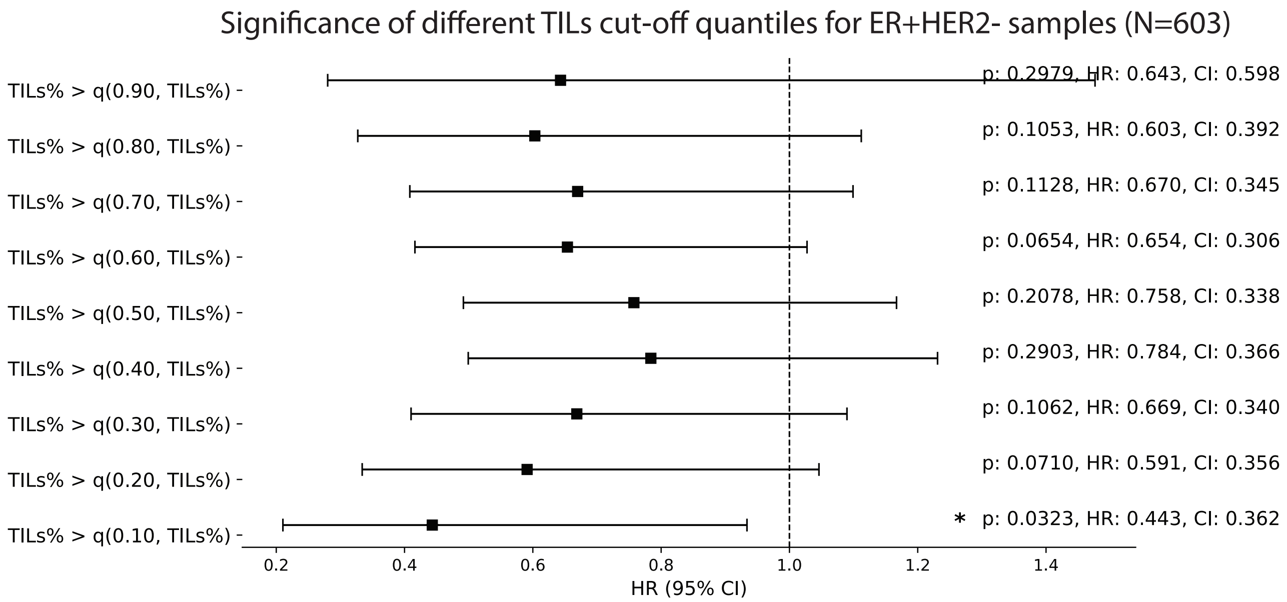
Significance of different TILs cut-off quantiles for ER+HER2- samples (N=603)
TILs% > q(0.90, TILs%)
TILs% > q(0.80, TILs%)
TILs% > q(0.70, TILs%)
TILs% > q(0.60, TILs%)
TILs% > q(0.50, TILs%)
TILs% > q(0.40, TILs%)
TILs% > q(0.30, TILs%)
TILs% > q(0.20, TILs%)
TILs% > q(0.10, TILs%)
p: 0.2979, HR: 0.643, CI: 0.598
p: 0.1053, HR: 0.603, CI: 0.392
p: 0.1128, HR: 0.670, CI: 0.345
p: 0.0654, HR: 0.654, CI: 0.306
p: 0.2078, HR: 0.758, CI: 0.338
p: 0.2903, HR: 0.784, CI: 0.366
p: 0.1062, HR: 0.669, CI: 0.340
p: 0.0710, HR: 0.591, CI: 0.356
* p: 0.0323, HR: 0.443, CI: 0.362
0.2
0.4
0.6
0.8
1.0
1.2
1.4
HR (95% CI)

Supplementary Figure 5: Exploring the TILs-AI cut-off in ER+ patients (METABRIC)

Supplementary Table 1. Average confusion matrix for the cell classification model over 5 fold

		Predicted				Avg. Acc.
		Fibroblast	Lymphocyte	Tumor	Other	
Ground Truth	Fibroblast	692.2	78.8	158.8	24.8	0.725
	Lymphocyte	83.6	918	127.2	6.8	0.808
	Tumor	89.4	124.4	5934.4	39.4	0.959
	Other	43.8	8.8	56.6	2033.8	0.949

Overall average accuracy	0.919
Normalized average accuracy	0.860

Supplementary Table 2. Clinical characteristics for spatial clusters in ICGC (proportions of total cohort)

	**All BASIS**	TIL-hi/Fibro-low (11.1%)	Fibro-hi/EMT (22.1%)	TIL-hi/Fibro-hi/Inflam(22.1%)	TumorDense(28.1%)	TumorDense/Oxidative (16.6%)	p-value $\chi2$ contingency
ER status							1.2e-3
Negative	**0.238**	0.500	0.096	0.288	0.258	0.154	
Positive	**0.762**	0.500	0.904	0.712	0.742	0.846	
N stage (Nx not reported)							0.473
N0	**0.349**	0.346	0.365	0.308	0.318	0.436	
N1	**0.238**	0.308	0.404	0.231	0.136	0.154	
N2	**0.094**	0.038	0.096	0.154	0.061	0.103	
N3	**0.017**	0.000	0.038	0.019	0.015	0.000	
T stage (Tx not reported)							0.776
T1	**0.255**	0.192	0.308	0.212	0.258	0.282	
T2	**0.357**	0.423	0.423	0.404	0.273	0.308	
T3	**0.085**	0.115	0.115	0.096	0.015	0.128	
T4	**0.034**	0.000	0.058	0.038	0.030	0.026	
Lymph Node							0.178
Negative	**0.507**	0.500	0.400	0.442	0.621	0.556	
Positive	**0.493**	0.500	0.600	0.558	0.379	0.444	
Grade							4.7e-4
1	**0.139**	0.080	0.163	0.174	0.115	0.143	
2	**0.454**	0.240	0.694	0.348	0.377	0.543	
3	**0.407**	0.680	0.143	0.478	0.508	0.314	
PAM50							0.024
Basal	**0.181**	0.389	0.051	0.235	0.186	0.143	
HER2	**0.045**	0.000	0.051	0.059	0.070	0.000	
LumA	**0.297**	0.222	0.513	0.294	0.140	0.286	
LumB	**0.445**	0.389	0.385	0.324	0.558	0.571	
Lympho- vascular invasion							0.646
Absent	**0.825**	0.800	0.776	0.800	0.860	0.886	
Present	**0.175**	0.200	0.224	0.200	0.140	0.114	
Lymphocyte infiltration							6.9e-5
None	**0.113**	0.001	0.128	0.111	0.082	0.229	
Mild	**0.632**	0.417	0.809	0.467	0.705	0.629	
Moderate	**0.189**	0.458	0.064	0.311	0.164	0.057	
Severe	**0.066**	0.125	0.000	0.111	0.049	0.086	
Tumor border							1.1e-4
infiltrative	**0.689**	0.591	0.826	0.795	0.627	0.543	
pushing < 50%	**0.097**	0.091	0.043	0.114	0.119	0.114	
pushing > 50%	**0.209**	0.318	0.130	0.068	0.254	0.343	
pushing and infiltrative	**0.005**	0.000	0.000	0.023	0.000	0.000	
Central scar/ fibrosis							0.070
Absent	**0.743**	0.783	0.652	0.837	0.661	0.857	
Present	**0.257**	0.217	0.348	0.163	0.339	0.143	
Necrosis							0.014
Absent	**0.777**	0.609	0.927	0.850	0.712	0.735	
Present	**0.223**	0.391	0.073	0.150	0.288	0.265	
TP53							0.031
Negative	**0.651**	0.423	0.769	0.596	0.652	0.718	
Positive	**0.349**	0.577	0.231	0.404	0.348	0.282	
MYC							0.327
Negative	**0.826**	0.808	0.904	0.788	0.773	0.872	
Positive	**0.174**	0.192	0.096	0.212	0.227	0.128	
PTEN							0.675
Negative	**0.838**	0.808	0.827	0.788	0.879	0.872	
Positive	**0.162**	0.192	0.173	0.212	0.121	0.128	
PIK3CA							0.953
Negative	**0.672**	0.731	0.654	0.673	0.682	0.641	
Positive	**0.328**	0.269	0.346	0.327	0.318	0.359	
PR status							2.4e-3
Negative	**0.319**	0.538	0.137	0.373	0.379	0.237	
Positive	**0.681**	0.462	0.863	0.627	0.621	0.763	

Supplementary Table 3. Clinical characteristics for spatial clusters in METABRIC (proportions of total cohort)

	**All METABRIC**	TIL-hi/Fibro-hi/Inflam (13.2%)	TIL-hi/Fibro-low (12.6%)	TIL-med/Fibro-hi/EMT (28.3%)	TumorDense/ Oxidative (29.2%)	Fibro-med/TIL-low (3.6%)	TumorDense (13.1%)	p-v $\chi 2$ contingency
ER status								0.012
Negative	**0.221**	0.231	0.202	0.154	0.267	0.167	0.287	
Positive	**0.779**	0.769	0.798	0.846	0.733	0.833	0.713	
Lymph Node								0.383
Negative	**0.476**	0.504	0.552	0.441	0.458	0.514	0.481	
Positive	**0.524**	0.496	0.448	0.559	0.542	0.486	0.519	
Grade								0.186
1	**0.111**	0.109	0.124	0.119	0.087	0.057	0.150	
2	**0.373**	0.403	0.331	0.418	0.332	0.486	0.354	
3	**0.516**	0.487	0.545	0.463	0.580	0.457	0.496	
TP53								4.0e-3
Negative	**0.886**	0.901	0.831	0.881	0.917	0.727	0.926	
Positive	**0.114**	0.099	0.169	0.119	0.083	0.273	0.074	
PAM50 (NC not rep.)								0.030
Basal	**0.174**	0.174	0.160	0.153	0.196	0.167	0.183	
HER2	**0.110**	0.129	0.104	0.085	0.113	0.056	0.160	
LumA	**0.334**	0.356	0.336	0.370	0.285	0.361	0.336	
LumB	**0.265**	0.182	0.328	0.178	0.347	0.278	0.290	
Chemo-therapy								0.159
Not treated	**0.715**	0.728	0.660	0.712	0.749	0.763	0.667	
Treated	**0.285**	0.272	0.340	0.288	0.251	0.237	0.333	
Hormone-therapy								0.0017
Not treated	**0.298**	0.352	0.223	0.341	0.309	0.382	0.139	
Treated	**0.702**	0.648	0.777	0.659	0.691	0.618	0.861	
HER2								0.2467
Negative	**0.801**	0.824	0.819	0.841	0.753	0.809	0.806	
Positive	**0.199**	0.176	0.181	0.159	0.247	0.191	0.194	
Lymphocyte infiltration								0.0095
None	**0.192**	0.284	0.123	0.202	0.194	0.270	0.172	
Mild	**0.636**	0.545	0.749	0.615	0.606	0.554	0.552	
Severe	**0.172**	0.171	0.128	0.183	0.200	0.176	0.276	

Supplementary Table 4. GSEA statistics output for the ICGC cohort. attached excel file)
Supplementary Table 5. GSEA statistics output for the METABRIC cohort. (attached excel file)

Supplementary Table 6. Overview of HER2 status in ER+ samples H&E slide dataset METABRIC

**Cluster**	**ER+, count (n=753)**	**ER+HER2-, count (n=630)**	**ER+HER2+, count (n=123)**
TIL-hi/Fibro-hi/Inflam	93 (12.4%)	82 (13.0%)	11 (8.9%)
TIL-hi/Fibro-low	99 (13.2%)	85 (13.5%)	14 (11.4%)
TIL-med/Fibro-hi/EMT	230 (30.5%)	197 (31.3%)	33 (26.9%)
TumorDense	209 (27.8%)	164 (26.0%)	45 (36.6%)
Fibro-med/TIL-low	30 (3.9%)	25 (3.7%)	5 (4.1%)
TumorDense/Oxidative	92 (12.2%)	77 (12.2%)	15 (12.2%)

Supplementary Table 7. Samples of the ICGC used for training of the model

Sample ID	Subtype
PD4267	ER+PR+HER2-
PD18257	ER+PR+HER2-
PD13627	ER-PR-HER2-
PD11816	ER+PR+HER2-
PD14435	ER+PR+HER2-
PD14437	ER+PR+HER2-
PD13631	ER+PR+HER2-
PD13622	ER-PR-HER2-
PD13620	ER+PR+HER2-
PD4072	ER+PR+HER2-
PD9539	ER+PR+HER2-
PD4986	ER+PR+HER2-
PD9579	ER-PR-HER2-
PD18251	ER+PR+HER2-
PD4959	ER+PR+HER2-
PD4962	ER+PR+HER2-
PD4965	ER+PR+HER2-
PD4967	ER+PR+HER2-
